# Sociocultural factors affecting first-year medical students’ adjustment to a PBL program at an African medical school

**DOI:** 10.1186/s12909-024-05229-0

**Published:** 2024-03-13

**Authors:** Masego B. Kebaetse, Dominic Griffiths, Gaonyadiwe G. Mokone, Mpho S. Mogodi, Brigid G. Conteh, Oathokwa Nkomazana, John Wright, Rosemary Falama, Maikutlo Kebaetse

**Affiliations:** 1 Department of Medical Education, University of Botswana, Gaborone, Botswana; 2 Wits School of Education, University of the Witwatersrand, Witwatersrand, South Africa; 3 Department of Biomedical Sciences, University of Botswana, Gaborone, Botswana; 4 Communication and Study Skill Unit, University of Botswana, Gaborone, Botswana; 5 Department of Surgery, University of Botswana, Gaborone, Botswana

**Keywords:** First-year medical students, PBL, Sociocultural, Adjustment, bi/multilingual

## Abstract

**Background:**

Besides regulatory learning skills, learning also requires students to relate to their social context and negotiate it as they transition and adjust to medical training. As such, there is a need to consider and explore the role of social and cultural aspects in student learning, particularly in problem-based learning, where the learning paradigm differs from what most students have previously experienced. In this article, we report on the findings of a study exploring first-year medical students’ experiences during the first semester of an undergraduate problem-based learning medical program at an African medical school.

**Method:**

We employed a qualitative case study approach using in-depth interviews with 23 first-year medical students. Participants ranged in age from 18 to 25 years. All students were bi/multilingual (some spoke three to five languages), with English as the learning language. We conducted an inductive thematic analysis to systematically identify and analyze patterns in the data using the Braun and Clarke framework.

**Results:**

Before medical school, students worked hard to compete for admission to medical school, were primarily taught using a teacher-centered approach, and preferred working alone. At the beginning of medical school, students found it challenging to understand the problem-based learning process, the role of the case, speaking and working effectively in a group, managing a heavy workload, and taking increased responsibility for their learning. By the end of the first semester, most students were handling the workload better, were more comfortable with their peers and facilitators, and appreciated the value of the problem-based learning approach.

**Conclusions:**

Our study highlights the importance of interrogating contextual sociocultural factors that could cause tension when implementing problem-based learning in non-western medical schools. Adjustment to problem-based learning requires a conceptual and pedagogic shift towards learner-centered practice, particularly concerning self-direction, the role of the case, and collaborative learning. As such, there is a need to develop and implement research-informed learning development programs that enable students to reflect on their sociocultural beliefs and practices, and enhance their regulatory learning competence to optimize meaningful and early engagement with the problem-based learning process.

## Introduction

The implementation of problem-based learning (PBL) is part of a broader shift toward a constructivist-oriented learning paradigm [1] learner-centered teaching [2], and, to some extent, the problem-posing model of education [3] partly intended to confront students’ apathy and lack of excitement and motivation about learning during medical school [4]. Learning challenges experienced by medical students have generally been associated with limited regulatory learning skills, such as poor study and critical thinking skills [5], challenges with metacognition and self-regulation [5], and the inability to transition to self-directed learning [6]. However, learning also requires students to relate to their social context [7] and negotiate such a context as they transition and adjust to medical training. As such, there is a need to consider and explore the role of social and cultural aspects in student learning [8–12], particularly in PBL, where the learning paradigm differs from what most students have previously experienced.

Sociocultural theory, emanating from Vygotsky’s work, provides a perspective for understanding students’ experiences with PBL in medical education beyond regulatory learning skills, in that human cognition and learning are developed through social activities. Thus, the “social and cultural context” of human cognitive development is critically important [13]. That is, learners do not operate “in a social vacuum but [are] part of a more complex community of practice, one in which they gradually gain access” [12]. For Vygotsky, there is a relational ontology between individuals and their world [14]. This situated, embedded relationality implies that any practice “is not ontologically separable from learning and human development, but the very substance of it” [15]. Sociocultural theory argues that learning modes, language practices, and relationships of authority will be context-dependent [16] Thus, to determine whether meaningful learning is taking place, it is necessary to account for and acknowledge the specific social and cultural contexts that an individual already inhabits.

Non-Western literature on the sociocultural aspects of PBL implementation generally relates to Asian [10, 17, 18] and Middle Eastern [19] students. On the contrary, a scan of African PBL literature of the past two decades showed that it primarily focuses on implementation [20], student and tutor perception of the PBL process [21–25], and comparisons of student performance in PBL and traditional programs [26, 27] with limited reference to sociocultural factors. The scarcity of African PBL studies addressing sociocultural aspects of PBL thus may pose challenges for medical schools seeking to implement PBL in African contexts characterized by multiple languages and subcultures. As Frambach et al. [28] argue, PBL is itself a “plural construct,” and thus, it is important “to learn from the cultural and situational nuances of educational activities labeled PBL around the world.”

As shown above, there is sufficient indication from the literature that sociocultural factors play a role in students’ adjustment to PBL. In this article, we report on the findings of a study exploring first-year medical students’ experiences during the first semester of an undergraduate PBL medical program at an African medical school. We reflect on these findings to gain insights into how socio-cultural factors related to our context might affect our students’ adjustment to PBL. The insights will shape a learning development program for first-year medical students in an African context.

## Materials and methods

We employed a qualitative case study approach [29, 30] to gain insight into our first-year students’ experiences with the problem-based learning program. This allowed us to thoroughly explore our students’ experiences while transitioning to a PBL program. This study was conducted at the end of the first semester of their medical training.

### Study setting and context

The Bachelor of Medicine Bachelor of Surgery (MBBS) program at the University of Botswana Faculty of Medicine (UB FoM) is a five-year undergraduate program. The UB FoM MBBS is a system-based, hybrid PBL program. A Presidential Task Force appointed in 1995 to assess the feasibility of Botswana establishing a medical school [31–33] determined that, with its expansive land mass and a significant population in rural and remote areas, Botswana’s health system needs doctors who can work independently across the country. While there are other student-centered active learning approaches, PBL was considered the core pedagogy that could train this kind of doctor, a perspective endorsed by the University senate when approving the medical school curriculum a decade later [34].

One or two weekly clinical cases drive the learning for each week [32]. In the first year, students learn about the normal structure and function of the human body and not about disease or disease processes. The use of clinical cases in PBL is to provide a learning context and relevant triggers for the learning objectives [35, 36]. Students start learning about disease (pathophysiology) in the second year. The program uses the Maastricht seven-jump process [35, 36] to facilitate the PBL small-group tutorials. PBL tutorials are complemented by other learning events such as self-directed learning, plenaries, bio-practicals, clinical placement, clinical and communication skills sessions, and resource sessions [32]. The plenaries are intended to clarify complex concepts and address students’ questions from various learning events [37].

The UB FoM MBBS program is not a direct entry program. Students are admitted through a highly competitive process after a year in a Bachelor of Science (BSc) or pre-med program or upon completion of the A-level program. The first two years focus on biomedical sciences, public health, basic clinical and communication skills, and weekly clinical placements [32]. In the first semester, students complete Foundations of Medicine (SoM 201), Cardio-Respiratory Systems (SoM 202), and Gastrointestinal and Urinary Systems (SoM 203). Anatomy is introduced from SoM 202 when students focus on specific systems.

### Study team

We formed a multidisciplinary team to explore (i) our students’ experiences in transitioning from the predominantly teacher-centered BSc program to the learner-centered PBL program and (ii) if the sociocultural factors of our context could affect student adjustment. The research team (four male and six female members) comprised biomedical scientists and educationists at master’s and Ph.D. levels, medical and public health specialists, and a fifth-year medical student. The team included a research consultant who participated in the study design and conducted all the interviews. Our shared belief was that understanding students’ learning challenges would enable us to develop better processes to support the students. Still, we lacked an in-depth, shared understanding of our students’ experiences in transitioning to a PBL program during the first year of medical training. However, given that most team members (i) shared a similar sociocultural background (i.e., were bi/multilingual, educated in the same teacher-centered public school system, and had gone through the same year-one BSc program at UB) as most of our students, and (ii) were PBL facilitators or familiar with the PBL process, we felt that we could relate to our students’ learning challenges and better support their adjustment to PBL. In this respect, we would act as role models for the students, encouraging them to trust the PBL process, advising them that the adjustment process would take time, and emphasizing the centrality of PBL cases as underpinning the learning process. This study prompted a greater degree of reflexivity by the team members, allowing us, through the data analysis, to understand and recognize the sociocultural challenges that our students had to overcome more formally. Similarly, the process highlighted the limitations in our PBL training of students and facilitators.

### Study participants

One of the investigators, who was not involved in teaching the students, invited all 51 first-year medical students (26 male, 25 female) in the cohort to participate in the study after a face-to-face class session. The interviewer followed up with interested students via phone calls and emails to set interview appointments. Twenty-four students agreed to participate, and all of them completed the study. One of the interviews was discarded as the recording was unusable, resulting in 23 interviews (from 11 male and 12 female students). Our sample size was within the recommendations by Morse [38] and Creswell and Creswell [39], who suggested a minimum of six or five to twenty-five participants, respectively. Alternatively, saturation may be used to determine the sample size when no more new information is generated [48].

The 23 students were 18 to 25 years old. Fourteen came from the BSc program, seven from the pre-med program, one student came both from A-levels and pre-med, and there was no data for one student. Three international students had previously attended private senior secondary schools, while all the local students had attended public senior secondary schools. Although all students used English as the language of instruction, all were bi/multilingual (some spoke three to five languages). Students reported that they mainly communicated in English with their peers, especially when the peers did not speak the native language and code-switched between English and the vernacular Setswana language. Most students stayed on campus (*n* = 15), a few stayed off campus (*n* = 2), and there was no data on the residential status for the rest (*n* = 6 ).

### Data collection

The consultant collected data through non-participant observations and three batches of in-depth semi-structured interviews. Interviewing in batches allowed us to pause and review the data. Observations and interviews were conducted at the medical school building on the UB campus. The findings of the non-participant observations contributed to development of the interview questions. Interviews generally lasted 90 min, were audio-recorded, transcribed verbatim, and checked against the audio to ensure accuracy. There were three focus areas for our questions, as shown in Table 1.

**Table 1:** Focus areas of questions for students’ interviews

Focus area	The focus of the questions
Focus area 1	Demographic information: academic background, some of the reasons for wanting to study medicine, and general experiences thus far with the MBBS program
Focus area 2	Overall impressions of the PBL process at this point in their studies
Focus area 3	Impression of PBL tutorials, including preparation, feedback, and challenging aspects

### Ethical considerations

After receiving a detailed explanation of the study’s procedures, risks, and benefits, the interviewer allowed all participants to review and sign the consent form. Participants were notified that they could withdraw from the study at any time without penalty. To ensure participants’ confidentiality and privacy, each transcript was allocated a code, e.g., G3S6, and any information that could be traced to the participants was de-identified, including presenting quotes anonymously in any reports and manuscripts. Data will only be kept for five years after the final publication from the study.

Ethical approval was granted by the University of Botswana Institutional Review Board (Permit# REF:UBR/IRB/1383).

### Data analysis

We conducted an inductive thematic analysis to systematically identify and analyze patterns in the data using the Braun and Clarke framework [40–42]. We each read and re-read three transcripts for familiarization, generated initial codes through open coding, met to discuss the codes, and reached a consensus [40, 41]. As we did iterative, multiple passes of reading the transcripts at this stage, we also listened for disparate voices while focusing on the whole account to get a good feel of the data. Subsequently, team members each read and coded at least two transcripts, while one team member read all the transcripts and consolidated the coding from the rest of the team. Next, we collated the various codes into potential themes and checked the codes to ensure that they were relevant to the theme and consistent with each other, checking themes for coherence. Then, we named and defined the themes. Given the nature of our data, a transitioning, narrative dimension emerged from the data during the inductive thematic analysis and became an essential part of the findings. For the scope of this paper, we focused on the themes and categories related to the two sociocultural factors.

## Results

In this section, we describe first-year medical students’ experiences as they journeyed through the first semester of medical school. We focus on students’ experiences (just before medical school, at the beginning of medical school [during the first three courses], and at the end of the first semester) with an undergraduate PBL medical program at a sub-Saharan African medical school.

### Students’ experiences just before medical school

The students’ journey to medical school started before enrolment. The participants generally described their learning experiences before medical school in terms of consistent hard work and high academic achievement: “I had to work really hard to know that I find myself a slot” (G3S2). The high academic achievement was complemented by a competitive process of achieving admission into the BSc/pre-med program and then medical school: “I did my first year as pre-med before I came here. […] we were in pre-med; we were competing to get into this side [medical school]. […] I was focused more on competing to get to this side” (G3S6).

In describing their prior learning experiences, participants chiefly characterized them in terms of the ‘banking model’ or teacher-centered approach. They described the teacher as the primary source of knowledge and themselves as recipients of the knowledge. This continued during the first year of university (BSc/pre-med), where they relied on listening to detailed lectures, receiving notes, and revising past examination papers: “But you kind of are [spoon-fed] in BSc. They give you everything, examples, and whatnot. All you have to do is just study on what they taught you. […] they literally teach you everything you need to know” (G3S8). Similarly, some students tended to learn with minimal effort, relying on their intellect and retention ability: “Yeah, I didn’t study a lot” (G1S5); or, as another student noted: “I didn’t have to study frequently at all” (G2S1).

### Students’ experiences at the beginning of medical school (during the first semester)

Participants described the MBBS learning environment as unfamiliar and challenging compared to their prior BSc/pre-med learning experience. It took many students time to understand the PBL process, as one student noted:

But problem-based learning, […] I honestly just didn’t know what it was. I was still getting to learn what it was about because there are steps to how to do PBL […]. At first, […] I just didn’t know how or what we were supposed to do. Once I got to see […] what I was supposed to do and how I got to learn, and how it’s related to our plenaries and stuff like that, then it made more sense, and then it was better (G3S8).

Four aspects of the PBL learning environment that students initially found unfamiliar and challenging are described below. These are *learning from an integrated PBL case*, *collaborative learning*, *substantial workload*, and *responsibility for learning*.

#### Learning from an integrated PBL case

Initially, students found learning from a case to be challenging as it required an integrated, holistic approach (drawing from multiple disciplines), unlike the more familiar disciplinary silo structure of BSc/pre-med. However, the situation improved as facilitators coached students by providing scaffolding structures for approaching cases: “The facilitator would say, ‘Think about the anatomy, think about the physiology, think about the public health’ […] it helped. It triggered the transition” (G1S4). In addition, all students indicated brainstorming (including developing objectives) as the most problematic aspect of the PBL process, as indicated by one student:

I don’t know if we were doing right or not doing right […]. I think until the end of the semester, we still have difficulty knowing exactly how we should write our problem and how they expect the brainstorming or the objectives we get from the brainstorming. But how the brainstorming should exactly be like and the problem, that’s [where] we had confusions (G3S1).

Another unfamiliar aspect of learning from a PBL case was learning the normal functions from the abnormal functions or ‘normal from the abnormal.’ Although students are presented with medical cases, some students understood the connection of learning the ‘normal from the abnormal’: “I think the case is more of pathology or abnormal state […]. Its aim is to make sure you know the normal state so that you can see how the abnormal comes from it […]” (G1S3). Another student noted: “If you’re dealing with a patient who has a problem with the intestines, you get to realize that I really need to know how the normal digestive system works” (G3S2). Some students did not seem to understand the connection between learning the ‘normal from the abnormal’: “We don’t know what exactly we are supposed to do. Are we supposed to do both normal and abnormal?” (G3S1). Even students who understood the connection acknowledged that, at times, it was challenging to learn from the abnormal when you do not know enough about the disease: “Getting to derive something normal from a disease which you don’t know about is quite challenging” (G1S3). Overall, the students described learning from the abnormal as not being problematic once they have more experience: “I don’t find it difficult [to relate normal physiology to pathophysiology]. I like relating things” (G1S2).

As they settled into the program at the end of the first six-week block, most students experienced the introduction of anatomy in the second block as a challenge. They used expressions such as ‘struggling’’, being ‘bad with anatomy,’ and ‘not knowing how to go about things.’ The students observed that anatomy entailed considerable detail: “Then we went to SOM 202 […]. I have problems with anatomy […]. I’m somehow finding it difficult to pay attention to those little details. Yes, because with anatomy, it’s all about knowing those little details” (G2S1). As such, the students acknowledged the need for a different approach to anatomy compared to studying physiology: “I studied anatomy like I was studying physiology. I will just read. But I had to be able to do it from the model or from the book, […] okay, fine, this is here. It’s this, and it’s here” (G3S1). However, unlike in physiology, there is a need for students to visualize body parts using models or their own bodies:

So afterward, I was able even to use myself as a model. I will look and say, ‘Okay, this is what I have. This is this.’ And then I think it was quite good […]. I don’t feel I look stupid, but everybody will be like, ‘What’s that girl doing?’ And I’m busy naming parts and saying stuff (G3S1).

Another student described going to the lab with peers to use models: “We had booked a lab at the same time. They also wanted to go to the lab for the models. When we got there, because we were a group, we started talking, discussing, discussing” (G2S1).

Students also suggested the need for an orientation to learning anatomy: “[…] just a way of saying, no this is anatomy, towards the left and right, just categorize these like this, and it will be much easier to think that way, not random bones and things, nerves all of it” (G1S1). The orientation included how to use the atlas, as one student recounted:

She did help me. Because, at first, I was like, ‘How am I going to study this big thing, the atlas?’ Then she just told me that the lab sheets, she gave us those to help us go through the atlas and it really helps. And it really helped me during the practical that we had because most of the stuff that was there was in those, in the lab sheets (G1S6).

#### Collaborative learning

The PBL curriculum is designed with collaborative learning as one of its core tenets. Almost all students described their preference for working alone and their initial reluctance to work in groups: “I’m old school […], I prefer to work alone” (G1S2). Another said:

I’m very much of a loner. I enjoy studying alone […], the person I can rely on most is myself. So, I do it my way […]. And it was working until I got here. So, I have to try and change, adapt, and I think I’ll get there (G1S5).

Initially, before students got used to one another and the PBL process, there tended to be silence in the tutorials, as the students found participating challenging. As one student noted, the challenge was, “Not being used to being in groups and discussing” (G3S4), or, as another observed: “For the first block we were just new. We were not yet comfortable” (G1S9). The lack of willingness to participate was worse for students who considered themselves ‘introverted’: “I prefer to listen and stay quiet, so having to speak was a challenge. I’m overcoming it. As a person, I have to motivate myself to speak up” (G1S4). Initially, some students were concerned about embarrassing themselves and appearing ‘stupid’ among their peers: “We didn’t know each other. Maybe if you say something, they will laugh” (G1S6); or, as another said: “When we are in a group where people are always above you [perform better than you], you feel you are low and it affects your contribution” (G1S9). Similarly, some students were unwilling to risk embarrassment in front of the facilitator: “If you ask, the facilitator will think this person hasn’t read or something” (G3S2); or, as another student noted: “Yeah, some people may say it is about respect [for the facilitator]” (G1S4).

For some students, the silence was also about the inability to frame questions properly: “I don’t think I’m good at […] structuring my questions. […] I can’t really simplify or make people understand what I’m, what do I really need to understand” (G2S6). The student further explained that English being a second language at times interfered with communication: “Some people just think they are not good at communicating. The language itself, English, is not the mother tongue so it can be a challenge. We just don’t know how to express or to say some of the things” (G2S6).

The students described some unproductive behaviors in the tutorial sessions during the early stages of medical school, including trying to ‘outdo’ one another in terms of responses without necessarily making meaningful contributions. Indeed, the data showed that sometimes peers were overly competitive, interrupting and not listening to one another, but merely being quiet long enough for the peer to stop talking so they could say something. Students also did not generally challenge one another’s thinking, partly because some students did not know how to receive feedback:

I don’t know if it’s all the time, but […] most of the time, we don’t listen to each other. […] It’s more of me saying what I have to score marks, […] not about me saying what I have so that people can understand. So, it will just be like, ‘Okay, fine, let him finish so that I can say my point and score my marks and go home.’ I think that’s all it is to it, and you don’t actually listen together. Because, even if you make a mistake or you say something that is not right, people can’t realize it. […] But I said something wrong, nobody is going to say anything (G3S1).

As the semester progressed and the tutorial group became more cohesive, the students described a change in both the silence and the dynamics of the group: “The silence has changed a lot […] as time goes on, you become open, you have created a bond” (G1S9). Or, as another student noted: “It [the silence in the group] has changed, it has really changed. You start knowing how to treat each other. So, we start to talk” (G1S6). By the end of the semester, nearly all the students were comfortable in their groups. As one noted: “I like my group. I’ve seen that they are very good people” (G1S3). In addition, they described the benefit of the tutorial group as providing opportunities to test their understanding of content and the best place to ask questions:

I like it when we exchange ideas […]. I’ll have a different take on a certain topic or a different understanding, and then someone will come up with a different view. So we kind of exchange views. So, you learn also in the process (G1S4).

Most students credited the small size of the tutorial group to creating comfort with peers and the facilitator, making it easier to participate than in the plenaries: “I don’t usually ask questions in class, more especially when we’re in plenaries […] when we are the whole class. But, in PBL I do ask questions because there is just a few […] of us” (G2S3). Furthermore, many students commented on how empathy had developed within their groups as they got to know each other: “You start knowing how to treat each other […] this one is sensitive, so we end here, this one I can go a bit further” (G1S6). Or, as another student said: “We don’t want to intimidate each other. I wouldn’t say ‘I think you’re wrong’ because I feel he or she will be offended or intimidated” (G1S7).

#### Substantial workload

The students described the workload as substantial, requiring many hours of studying: “The amount of material we covered in 201, ah, it amounts to the whole of BSc [laughing]. I tell you, that was the whole of BSc” (G2S4). They described the workload as overwhelming and stressful, using expressions such as “it’s really stressful” (G1S5), “I was stressed out. I was really stressed out” (G2S1), “a lot of pressure” (G3S2), and “I was too pressured. Actually, at first, I was so freaked” (G2S3). Some students were disappointed that their marks did not match the effort they put into studying, although the low grades did not dissuade students from hard work, as hard work was needed to ‘maintain’ grades and ensure that their grades did not worsen. The students also expressed concern that the medical school took up so much time that there was little time left for anything else: “It’s just a whole lot of material that you have to do in a short space. So, that on its own really puts pressure on us. We really don’t have time for anything other than books” (G2S3).

#### Responsibility for learning

The students discussed having to assume increased responsibility for their learning and having to be self-disciplined, self-directed, and self-reliant:

So here, you have to self-motivate […]. You don’t get motivation from your lecturers […]. So, you get to have self-responsibility, drive your own things, make your own life timetable, know what to do at what time […]. I just learned, like, self-responsibility (G3S2).

Plenaries in the MBBS program provided “an overview of the topic, and then you have to do most of the reading at home” (G2S3) to cover the necessary objectives. The students had to do a considerable amount of independent learning:

[…] usually, in the lectures, we don’t get deep into stuff […], we only go shallow. So, PBL helps us, […] we do come up with some learning objectives related to the biomedical lectures and the PBL. So, we go research on that, and we’d be […] learning deeper (G2S7).

As the teacher is no longer the sole knowledge authority, successful learning requires focus and good study habits. In addition, learning effort is critical, as “it’s no longer about how smart you are. The level of intelligence got you here, but the work you put in makes you stay here” (G2S2). Furthermore, most students reported changing their study strategies to accommodate the workload, for example, by increasing study time and adhering to a schedule.

### Students’ experiences at the end of the semester

Towards the end of the semester, most students were settling into the program, and all reported enjoying the program despite the workload and challenges. At this point, most had a clearer understanding of the PBL process and appreciated the value of the PBL approach.

#### Views on the enjoyment of the program

Despite the substantial workload, achieving lower marks than in BSc, and the unfamiliarity of the PBL approach, all students reported enjoying the program: “Despite the marks I get, I’ll say, yes [laughs] I think I like the challenge mostly. […] And the people I’m always with, they make it more enjoyable” (G1S5). A few, however, described liking the challenge and pressure of medical school: “[…] I’m enjoying the pressure more than anything else [laughing]” (G3S1). Some also enjoyed learning something new every day: “Apart from the fact that medicine is interesting on its own, every day is a learning curve for you. You grasp almost a new concept all the time” (G2S5). In addition, clinical placements added to the enjoyment of the program in that they exposed students to how life would be as a doctor: “Every Wednesday, we go there [clinical placement], so [it is] always exciting. Just the fact that we get to […] see first-hand what we’re supposed to be doing” (G3S3).

#### Views on the benefits of PBL

The students also appreciated and recognized some benefits of PBL. Even those who did not like the PBL approach acknowledged that the opportunity to speak and present among peers was an advantage: “Well, for me, it [PBL] hasn’t contributed that much […] except that maybe I can talk to people, something that I didn’t do in the past. […] That’s all, I think, the positive part of it” (G1S5). Furthermore, being confident enough to speak in a group suggests that they had to learn and understand what they were presenting: “If I’m able to voice it out here, it means I learned a whole lot about it” (G3S3). Another benefit that was highlighted related to independent learning and coming back to share with peers:

I think PBL is really helpful […] considering that you go out on your own, you research. […] Sometimes you just go an extra mile in PBL, and going that extra mile, it kind of lightens up, it shows things to you […]. And now having to be with people around you and they introduce stuff that you didn’t know, or the stuff that you skipped, and there they explain that. […] So, it’s really interesting (G2S1).

#### Appreciating the value of the PBL approach

As the first semester ended, most students described a better understanding of the PBL process and recognized that the case and tutorials were at the core of the learning process. They realized that in attending the plenaries and practical sessions and utilizing the objectives from the block guide and the case for self-study, they were equally preparing for the feedback tutorial, tests, and examinations:

At first, it was a little bit tough because […] we didn’t have the strategy to make sure that there is an organization between the PBL, the lectures, and your studying. So, these days, we managed to try and organize so that […] it becomes easier. […] I realized that […] the PBL is not like it’s something separate […] it’s related to the material for the lectures in the content for objectives for that week. So, when I realized that, I just take these objectives that we are given in PBL, I make sure it’s part of my revision. I no longer see there is PBL, there is revision, no. When I’m doing PBL, I’m revising, and I’m also doing […] part of the lectures. I think it’s a cornerstone (G1S9).

However, even at the end of the semester, some students still isolated the learning events, and they still spoke of ‘doing PBL’:

We do have objectives on Monday, so most of the times we have two cases. What I’ll simply do, I’ll take one on Tuesday and then the other one on Wednesday. So, I don’t do PBL at my studying time. […] Mostly on Wednesdays, we have clinical placements, so I would go there maybe in the morning and come back around 13:00. Then, from there, I’ll take time to do my PBL after lunch at home. I’ll just do my PBL then. One case per day (G2S3).

### Summary of results

*Just before medical school*, students worked hard to compete for admission to medical school, were taught using a teacher-centered approach to learning, and preferred working alone. *At the beginning of medical school*, students had difficulty understanding the PBL process, the role of the case, speaking and working effectively in a group, managing a heavy workload, and taking increased responsibility for their learning. *By the end of the first semester*, most students were managing the workload better, were more comfortable with their peers and facilitators, and appreciated the value of the PBL approach.

## Discussion

Through this study, we add to PBL literature situated in a non-Western context by exploring how students’ experiences could be understood not just from the lack of regulatory learning skills, but also from a sociocultural perspective through pedagogical transitioning. Consistent with the thinking of Frambach et al. [28], we believe that medical schools should interrogate the sociocultural factors embedded in their contexts, as part of the effective implementation of PBL in non-Western contexts. As such, we discuss our findings considering two significant sociocultural factors contextualized in an African setting that may conflict with effective PBL implementation: the socialization of schooling before medical training and the cultural socialization relating to speaking and silence.

*The socialization of schooling before medical training* involves students entering medical training with beliefs about teaching and learning established through a history of high academic achievement. For some students, these beliefs are steeped in a predominantly teacher-centered or ‘banking model’ of educational orientation, where the teacher is the ‘narrator’ and students are ‘containers’ that receive and store information [3]. Although concerns have been raised about teacher-centered approaches [43], learning during the BSc program was essentially a continuation of the teacher-centered approach in which our students had succeeded academically through the years. The first year of the BSc program allowed students to operate within a familiar socialization of schooling that did not conflict with students’ sociocultural socialization. This included the teacher as the primary source of information, a proclivity for academic competitiveness, and a preference for working alone. It is unsurprising that our students, who were high academic achievers to start with, continued to perform well at this stage. It has been suggested that most Batswana students (i.e., citizens of Botswana) come from secondary schools where “the pedagogy used never attempted to develop independent thought, group activity or questioning of authority” [43]. This model of education leads to passive and non-responsive students, mainly when concepts are unclear, and students are afraid to ask questions [43].

The banking model orientation to schooling can create tension and compete with the self-directed learning required for a PBL curriculum [44, 45]. A PBL curriculum requires students to be strategic, self-regulating learners who assume increased responsibility for their education by planning and executing their learning plan, seeking and evaluating information, assessing their learning, and making the necessary changes [46]. While successful learning requires effort from the teacher and the student, students socialized predominantly through teacher-oriented practice may not value their own role in the learning equation [47].

In contrast to their prior learning, the introduction of PBL and the heavy workload associated with medical training initially creates a disequilibrium for the students. As previously suggested [48–50], our students found the initial experience with PBL challenging, primarily because of the tension between students’ beliefs and practices about teaching and learning in the teacher-centered, *and* the PBL approaches. In our context, and consistent with findings about non-Western students [9], one possible explanation could be the initial tension between the students’ sociocultural socialization, and the values inherent in self-direction and self-regulation as essential components of PBL. We think that the poor self-regulation (for example, lacking a learning strategy and insisting on being ‘taught’ and given notes) demonstrated by some of our students and consistent with previous literature [48], is a consequence of years of teacher-centered pedagogical approaches.

Poor self-regulation has been associated with stress and burnout [51], which may explain our students’ expressions of being ‘pressured,’ ‘stressed,’ ‘pulling me down,’ and ‘freaking out.’ We think that the nature of our hybrid PBL program could also contribute to a delay in students’ transition to self-direction and self-regulation. For example, some weeks have as many as 11 plenaries, and some plenaries have over 50 slides; these factors continue to reinforce the teacher as the primary source of information, and thus may encourage students to hold on to their prior socialization of schooling. These problems underscore the need for the teaching staff and curriculum implementation structures to understand and commit to the ethos of the PBL approach.

*The cultural socialization relating to speaking and silence* means that students often bring prior schooling cultural practices and expectations that interfere with efficient learning in PBL, particularly in tutorial groups. Students who prefer to work alone are confronted with a process where small-group learning is at the core of learning, and they need to develop collaborative learning skills of listening, questioning, and claiming their voices in the learning process [2, 52]. While PBL requires students to “show rather assertive behaviors, such as speaking up, asking questions, and challenging the opinions of others” [52], non-Western students may instead demonstrate non-engaging behaviors concerning speaking and silence that can inhibit their ability to question and engage in tutorial groups [10]. These practices are counterproductive to the benefits intended to be derived from small-group learning. For instance, amongst Asian students, silence may often be perceived as indicating proper manners or respect in the presence of elders or people of authority [10, 53]. This could be part of the perceived power or authority structures between the teacher and the student [3, 10, 53], students’ conception of their identity as learners [10, 53], or limited communicative competence [53].

Although collaborative learning is at the core of PBL [8, 54], some of our students, like other non-Western students [3, 10, 53] experienced challenges with speaking and silence within small groups. While models of group dynamics [55] may be used to explain speaking and silence within small groups, our results suggest that it may also be important to consider sociocultural factors of speaking and silence as an additional significant perspective. In the Botswana context, cultural practices and expectations embedded in Setswana identity could initially make Batswana students unable to participate meaningfully in PBL tutorials. This especially relates to silence as a form of communicative behavior [56]. As Bagwasi [56] argues, this interconnectivity between language and culture is reflected in the Setswana belief system, perceptions, behaviors, and speech acts. Central to these factors is the principle of *Botho*, which is a Setswana word for “respect, good manners, and good character” [43]. In the Setswana culture, silence can indicate subordination and politeness in relationships between children and adults and between men and women [56]. Children especially are expected to “remain silent in the presence of adults as a sign of subordination, respect, and humility” [56], placing children at the bottom of the social strata and socializing them not to be heard [56]. This cultural practice could pose challenges when students, who have traditionally been socialized to be silent, are suddenly expected to have a voice in the learning process. Furthermore, whereas in Western culture, it is understood that one may respectfully interrupt a speaker to express a contrary view, in Setswana, interrupting, or “*go tsena ganong*” (getting into the mouth of the speaker) [56] is viewed as poor manners and disrespectful. This predisposition may limit the possibility of having an active and engaging discussion, and could thus contribute to some students deferring to talkative students and remaining silent.

It is also possible that besides cultural practices and expectations, the culture of schooling regarding the use of English as the primary language of instruction poses challenges for students entering PBL. Although Botswana is a multilingual country with 28 languages, English is the official language of instruction from the second grade of elementary school [57]. English language proficiency, based on the national secondary school exit written examinations, is required for admission to the University of Botswana (UB). Additionally, upon entry to the UB, all students undertake communication and study skills courses to further develop their English language proficiency and help prepare them for university learning.Please connect the next paragraph here

However, in most of their pre-medical schooling, students learn in English, with an emphasis on English language proficiency through written communication. This can initially pose challenges for students entering PBL, where oral communication is the primary mode of learning in tutorial groups. For instance, students may be self-conscious about speaking, especially when contending with multilingualism in a monolingual classroom. Silence in tutorial groups may also be a symptom of challenges with self-expression in English [43, 49]. Thus, it is unsurprising to find some students reporting challenges with self-expression in English because English is not their native language, *and* they are learning the new and difficult language of medicine. Despite English being the language of instruction through most of their schooling, many of our students often code-switch between English and Setswana, reflecting a common use, if not accepted practice in our culture, including teaching and learning [50, 58–60]. Mokgwathi and Webb [58] have suggested that this culture of code-switching could be the reason for challenges with oral communication. One of the challenges with code-switching is that it may compromise the development of “learners’ proficiency and confidence in speaking English” [58]. Giving students additional opportunities to engage in collaborative learning in a non-threatening environment outside the PBL tutorial group could help their adjustment with respect to speaking and silence.

 Challenges notwithstanding, most students ultimately embraced the need for a conceptual change [61, 62] and pedagogic shift towards PBL [61, 62] as a learner-centered approach, replacing what did not work with what worked for the new learning environment. This pedagogic shift facilitated the students’ realization of the centrality of the case as driving all learning, placing greater value on independent learning and learning in groups, and managing their workload better. For these students, adjusting to the PBL approach was an evolution, not a revolution, consistent with a conceptual change process that requires time for intellectual development [54] and learning new habits and strategies. However, the proportion of those who fail because they are struggling with the learner-centered approach but would otherwise thrive in a teacher-centered approach is unclear. Hence, there is a need for adequate support and resources for all students to facilitate and enable them to be proactive in learning, as previously proposed [54].

### Study limitations, strengths and future research

Our findings cannot be generalized because they apply to our specific context. We also did not include the PBL facilitators’ perspectives for the scope of this paper, which could shed more light on our medical students’ learning challenges. The strength of our paper is that most African and perhaps other non-Western medical schools may relate to our findings. Furthermore, the paper highlights that medical schools globally should consider sociocultural factors that may conflict with PBL implementation.

Future manuscripts from our data will draw from theories of identity formation, transition, and communities of practice to generate insights into students' learning experiences as they adjust to a PBL curriculum in the context of African medical school.

## Conclusions

Our study highlights the importance of interrogating contextual sociocultural factors that could cause tension when implementing PBL in non-western medical schools. As such, there is a need to develop and implement research-informed learning development programs that enable students to reflect on their sociocultural beliefs and practices, and enhance their regulatory learning competence to optimize meaningful and early engagement with the PBL process.

## Data Availability

The interview transcripts can be requested from the corresponding author at kebaetsem@ub.ac.bw.
